# Ecological Niche Models and Coalescent Analysis of Gene Flow Support Recent Allopatric Isolation of Parasitoid Wasp Populations in the Mediterranean

**DOI:** 10.1371/journal.pone.0005901

**Published:** 2009-06-12

**Authors:** Jeffrey D. Lozier, Nicholas J. Mills

**Affiliations:** 1 Department of Entomology, University of Illinois, Urbana, Illinois, United States of America; 2 Department of Environmental Science, Policy and Management, University of California, Berkeley, California, United States of America; University of Bristol, United Kingdom

## Abstract

**Background:**

The integration of multiple complementary approaches is a powerful way to understand the processes of diversification and speciation. The parasitoid wasp *Aphidius transcaspicus* Telenga (Hymenoptera: Braconidae) is a parasitoid of *Hyalopterus* aphids across a wide geographic range. This species shows a remarkable degree of genetic structure among western, central, and eastern Mediterranean population clusters. In this paper we attempt to better characterize this genetic structure.

**Methodology/Principal Findings:**

We use a Bayesian coalescent analysis of gene flow under the *Isolation with Migration* model using mitochondrial and microsatellite markers together with climate-based ecological niche models to better understand the genetic structure of *A. transcaspicus* in the Mediterranean. The coalescent analysis revealed low levels of migration among western and eastern Mediterranean populations (*Nm*<1) that were not statistically distinguishable from zero. Niche models showed that localities within population clusters each occupy areas of continuously high environmental suitability, but are separated from each other by large regions of completely unsuitable habitat that could limit dispersal. Overall, environmental characteristics were similar among the population clusters, though significant differences did emerge.

**Conclusions/Significance:**

These results support contemporary allopatric isolation of Mediterranean populations of *A. transcaspicus*, which together with previous analyses indicating partial behaviorally mediated reproductive isolation, suggest that the early stages of cryptic speciation may be in progress.

## Introduction

Working to understand the processes that contribute to reproductive isolation and speciation is among the most active and challenging areas in evolutionary biology. Over the last several decades the use of genetic markers to identify evolutionarily distinct populations has become common practice [1], with phylogeographic studies having demonstrated that any number of biotic and abiotic components—including geological, climatic, and ecological processes—can generate population structure and eventually lead to reproductive isolation and speciation. Studies of recent divergences are particularly attractive because the signatures of such events have not been fully erased by time, and it can be more straightforward to infer process from patterns in genetic data [2]. As phylogeographic studies accumulate, however, it is becoming clear that complete explanations of evolutionary history often require the integration of multiple complementary sources of information [3].

One powerful way to improve genetic inferences about gene flow and recent speciation is through the incorporation of environmental data [3]–[7]. Environmental variables determine, in part, the geographic distribution of a species as well as how selective pressures vary within that distribution, shaping the potential for and mechanism behind reproductive isolation. For instance, allopatric genetic differentiation occurs when populations become spatially separated by regions of environmentally unsuitable habitat, resulting in an interruption to gene flow and the gradual accumulation of reproductive isolation mechanisms unless the organisms adapt to the intervening inhospitable conditions [5]. Alternatively, for differentiation in sympatry or parapatry, geographic ranges of diverging populations may overlap or abut, but divergent ecological pressures within the overall distribution are more likely to drive the formation of reproductive isolation. However, even under allopatric conditions ecology can play a role; if isolated populations experience unique selective pressures then local adaptation and niche diversification may ensue, helping to reinforce reproductive isolation even if physical barriers to dispersal eventually disappear [8]. Understanding how environmental variables contribute to the geographic distributions of species is thus of considerable interest.

Geographic information systems based ecological niche modeling is a promising tool for predicting species distributions and identifying factors that have contributed to their divergence [5], [9], [10]. Such models divide the Earth's surface into a series of grid cells and, provided with coordinates of sampling localities and a set of spatially explicit environmental data layers, statistically assessing the suitability of each grid cell for the taxa of interest. These values are then used to generate a map predicting areas likely to be suitable for a species given the environmental variables included in the model. Results from ecological niche models (ENMs) can be integrated with hypotheses from molecular data to assess whether populations that show strong genetic structure are also likely to be geographically discrete (i.e., each occupying areas of high environmental suitability but with intervening areas of low suitability). The combination of such evidence would suggest that gene flow is likely to be effectively negligible and lineages may be in the early stages of speciation [5], [11], [12].

Once populations are shown to be structured both genetically and environmentally, a rigorous assessment of gene flow levels is needed to determine their evolutionary status and potential for speciation [1], [3]. While phylogenetic analyses or strong differentiation observed using population genetic statistics (e.g. *F*_ST_) may allow identification of evolutionary significant units, these approaches can be problematic for quantifying gene flow under recent divergence because they do not adequately separate the historical effects of shared ancestry from contemporary gene flow [13], [14]. However, new computational methods that explicitly model scenarios involving population divergence and ongoing gene flow using the coalescent process [15] can estimate such demographic parameters independently using multilocus genetic data. These methods can thus provide insights into both past and present processes and allow users to statistically distinguish the probability of current gene flow versus complete isolation [16]–[18].

**Figure 1:**
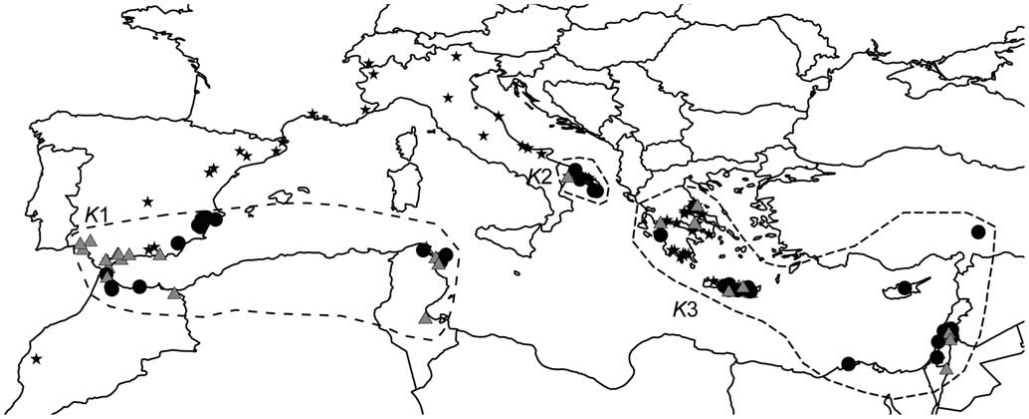
Map of *Aphidius transcaspicus* sampling localities in the Mediterranean region. Black circles represent localities from which microsatellite genotypes are available, gray triangles represent localities where *A. transcaspicus* was sampled but not genotyped, and black stars represent localities where *Hyalopterus* aphid hosts were abundant but *A. transcaspicus* was absent. Areas corresponding to the three genetic clusters *K*1−*K*3 identified in Lozier *et al*. (2008) are encompassed by dashed lines.

We undertook this study in an attempt to explain the remarkably high levels of genetic differentiation previously observed among population clusters of the wasp *Aphidius transcaspicus* Telenga (Hymenoptera: Braconidae) [19]. *A. transcaspicus* is a specialist parasitoid of aphids in the genus *Hyalopterus* Koch and is distributed throughout much of the Mediterranean and parts of central Asia. Bayesian cluster analyses of microsatellite markers have shown that individuals in the Mediterranean region are grouped into three main population clusters (*K*1−*K*3; Figure 1) that show striking levels of divergence, as measured using hierarchical *F*-statistics [20] (among-cluster *F*_CT_ = 0.45) [19]. Plots of isolation by distance show that over the geographic areas covered by each population cluster there is no increase of genetic divergence with geographic distance, but genetic distances for inter-cluster locality pairs are much greater [19]. Mitochondrial DNA (mtDNA) sequences from the same regions are not reciprocally monophyletic among population clusters, but still have very high levels of differentiation (overall *F*_ST_ = 0.68) and a unique set of haplotypes is restricted to parasitoids from *K*3 localities in the eastern Mediterranean, supporting the distinctness of this population [19]. Furthermore, laboratory mating experiments conducted using individuals collected from Spain and Israel (representing *K*1 and *K*3, respectively) suggest that this genetic differentiation may be correlated with limited reproductive compatibility between parasitoids from the different regions [21] (Lozier & Mills in prep.). While these experiments identified no obvious post-mating isolation effects in terms of offspring production, reciprocal inter-population mating either took substantially longer than intra-population crosses or were avoided entirely, suggesting that pre-zygotic isolation barriers, perhaps involving mate-recognition or courtship behavior [e.g. 22], have begun to evolve. Together, the genetic and behavioral information indicate that *A. transcaspicus* populations in the Mediterranean may be in the early stages of speciation.

Sampling data and field observations suggest that *A. transcaspicus* is most abundant in coastal Mediterranean areas, and becomes rare or absent above 42–43°N latitude in Europe and away from the coast in northern Africa. In contrast, its *Hyalopterus* hosts appear to have a much broader distribution, especially in Europe, where *Hyalopterus pruni* occurs as far north as England, Germany, Finland, and Sweden [23] (N Mills, personal observation). In this paper we use ENMs to investigate whether climatic variables may be limiting the geographic distribution of *A. transcaspicus* in the Mediterranean region and thus contributing to genetic differentiation at the region-wide scale through environmental barriers to gene flow. At the same time, the broad geographic area inhabited by *A. transcaspicus* may provide opportunities for ecological specialization in subdivided populations, and we assess this possibility by testing for differences in climatic variables experienced by parasitoids from each genetic cluster. To supplement this analysis, we reanalyze mitochondrial sequence and microsatellite data from Mediterranean populations [19] by fitting them to a coalescent-based *Isolation with Migration* (*IM*) model [16], [17], which estimates demographic parameters in recently diverged populations and allows the use of likelihood ratios to test the significance of nested models that include or exclude gene flow.

## Methods

### Genetic Analyses

We used genetic data from a previous study [19] to extend our analyses of the population structure of *A. transcaspicus* in the Mediterranean. Briefly, these data consist of a 432 bp segment of the mitochondrial *cytochrome oxidase I* gene (*COI*) and nine microsatellite loci [see 19], [24 for details and GENBANK accession numbers]. In our previous study, we genotyped parasitoids from most localities at which *A. transcaspicus* was observed (Figure 1) and found that Mediterranean *A. transcaspicus* are organized into three highly differentiated population clusters based on microsatellite genotypes: a western cluster comprising parasitoids from Spain, Morocco, and Tunisia (*K*1), a cluster from Italy (*K*2), and an eastern cluster comprising parasitoids from Greece, Cyprus, Egypt, Turkey, and Israel (*K*3). The *COI* data provided less resolution, but one set of haplotypes was found only in localities within the *K*3 cluster, highlighting the uniqueness of parasitoids in the eastern Mediterranean.

Our present analyses focus on quantifying the degree of differentiation among these three clusters by fitting our data to the two-population *Isolation with Migration* (*IM*) coalescent model as implemented in the IMa software [17]. The model assumes a panmictic ancestral population of effective size N_A_ that, at time t in the past, diverged into two daughter populations of sizes N_1_ and N_2_ that have undergone regular gene flow at a rate of m_1_ and m_2_ since divergence. IMa utilizes a Bayesian Markov Chain Monte Carlo method to estimate posterior distributions of the six demographic parameters in the *IM* model, scaled by the mutation rate μ, including the population size parameters (for haplodiploid organisms θ_A_ = 3N_A_μ, θ_1_ = 3N_1_μ, and θ_2_ = 3N_2_μ), the divergence time parameter (*τ* = tμ), and the post-divergence migration parameters (*m*_1_ = m_1_/μ and *m*_2_ = m_2_/μ, where, here, m equals the migration rate per generation *into* the specified population, considered *forward* in time). Upper bounds for the prior parameters were set at θ_1,2_ = 4, θ_A_ = 100, *τ* = 4, *m*_1_ = 10, and *m*_2_ = 10. These bounds were selected following preliminary runs with very large upper bounds followed by modification in subsequent trial runs depending on the resulting posterior distributions, as suggested in the IMa manual. For the final analysis, the program was run using 50 chains for a total of 5×10^7^ steps (sampling every 100) following a burn-in period of 500,000 steps. Following multiple trial runs, geometric heating parameters were chosen to achieve sufficient mixing (h1 = 0.99, h2 = 0.70) such that no obvious trends were apparent in posterior distribution scatter-plots. The IMa analysis is computationally intensive, and can thus only analyze a two population model. Therefore, we attempted to perform three analyses, one for each pairwise comparison between *K*1, *K*2, and *K*3. We used the full set of mtDNA sequences from each cluster (*n* = 51, 12, and 96, respectively) under an infinite sites evolution model, and a subsample of 100 randomly selected individuals each from *K*1 and *K*3 (all 17 genotypes for *K*2 were used) from each of five loci (At001, At003, At004, At009, At016) that did not obviously deviate from the stepwise mutation model (i.e. contained no allele sizes deviating from expected given the repeat unit). Unfortunately, convergence could not be achieved for comparisons involving *K*2 despite multiple attempts and so we focus here on the distinctiveness of the larger *K*1 and *K*3 clusters. The analysis was repeated for three short runs using different random seed values and visually assessed for consistency before performing the final run described above.

To ensure that posterior parameter distributions were scaled properly, inheritance scalars were assigned to each locus to account for the haplodiploidy of *A. transcaspicus* (diploid females, haploid males). Because IMa is designed to return estimates given a value of θ equal to twice the effective number of nuclear gene copies (in this case 1.5×N_e_), multiplied by μ, the nuclear microsatellite loci were given an inheritance scalar of 1.0, while the maternally inherited haploid mtDNA locus was given an inheritance scalar of 0.333 (J. Hey, personal communication). We report estimates for all parameters scaled by μ (but see Discussion).

We used 90% highest probability marginal-density (HPD) to examine the variance of parameter estimates. We also used the nested models approach implemented in the “L-mode” of IMa (using 5×10^5^ trees) to statistically test the hypothesis of zero gene flow for both *m*-parameters as compared to the full model containing all parameters using log-likelihood ratio tests. Because in our alternative hypothesis *m* is bounded by zero, to test the significance of no gene flow we used the following approach, recommended by J. Hey (internet communication on 26.iii.2007; http://groups.google.com/group/Isolation-with-Migration). First we tested whether the hypothesis of equal gene flow could be rejected (H_0_: *m*_1_ = *m*_2_ versus H_A_: *m*_1_≠*m*_2_, with all other parameters in the model free to vary) where the −2LLR statistic was tested by comparing maximum posterior probabilities for each model using a χ^2^ distribution with 1 d.f. The second test compared the −2LLR of H_0_: *m*_1_ = *m*_2_ = 0 versus H_A_: *m*_1_ = *m*_2_, using a mixed distribution of 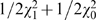 [17] implemented in the R 2.6.2 [25] package emdbook [26]. If neither test returns a significant result, then the hypothesis of *m*_1_ = *m*_2_ = 0 cannot be rejected, providing no support for gene flow levels greater than zero.

### Ecological Niche Model

#### Aphidius transcaspicus locality data

Museum and literature records can be useful tools for obtaining locality data for niche modeling [4], though are reliable only if specimens have been accurately identified. Because *A. transcaspicus* has a long history of taxonomic confusion [27]–[29], we suspected that many older historical records were potentially error-prone and chose to rely largely on our own extensive collections of the target species. Between 2002 and 2007— largely in May and June—field trips to Spain, Morocco, Tunisia, Italy, Greece, and Israel were conducted to obtain *A. transcaspicus* samples for importation to California, USA as part of a biological control program for the aphid *Hyalopterus pruni*. As both *A. transcaspicus* and its host aphids are sporadically distributed among host plants throughout the region samples were taken opportunistically by looking for *Hyalopterus*-infested *Prunus* trees or *Phragmites* plants (both both *Hyalopterus* host plants) and collecting any parasitized aphids found. In some countries samples were collected in more than one year during this period, for others only a single year of sampling was conducted. In total, *A. transcaspicus* was collected or observed at 70 locations throughout these regions (Figure 1). We also obtained specimens confirmed as *A. transcaspicus* from Cyprus, Egypt, Turkey, Iran, and Pakistan from collaborators (M. Hadjystilli, University of California at Berkeley; P. Starý, Czech Academy of Sciences, Czech Republic; E. Rakhshani, Tarbiat Modarres University, Iran). The four localities from Iran and Pakistan were used as training data for the ENM but are not discussed further. Finally, we used three localities in Greece obtained from a recent review of European *Aphidius* species [30], leading to a total of 81 records (Table S1). All localities were georeferenced from label data or field notes using regional maps and Google Earth. We note that areas where *Hyatlopterus* occurs outside of our ‘presence localities’ have been searched on several occasions for *A. transcaspicus* (e.g. France, Germany, northern Italy, Switzerland, northern Greece and coastal Baltic states), but no evidence of the parasitoid was found, despite an abundance of their aphid hosts (Figure 1; N. Mills, personal observation). Other recent surveys of parasitoids by *Aphidius* taxonomists familiar with the collection and identification of *A. transcaspicus* have also suggested a restriction to the “Mediterranean” faunistic complex, as opposed to boreal, steppe, coniferous, or deciduous habitats [29].

#### Modeling approach

We generated an ENM for *A. transcaspicus* using the robust modeling approach implemented in the program Maxent v3.1 [31]. Maxent uses presence-only sampling data and environmental data layers to generate a probability distribution of species occurrence over a given area using the principle of maximum entropy [31]. Our intention is to generate a distribution of geographic areas that are likely to be climatically suitable for *A. transcaspicus* in a typical contemporary environment, rather than predict on a fine-scale the occurrence in a given community or landscape. Although presence-only data is collected from a geographic area that represents the contemporary realized niche (α-niche) of a species, which may be reduced in size by biotic interactions from the area representing the true fundamental niche (β-niche) defined by climatic tolerance, this is of less concern for *A. transcaspicus*. As noted above, the distribution of *A. transcaspicus* is not limited by the distribution of its food resources (also see Discussion), and as both aphidophagous competitors and hyperparasitoids are also more widely distributed than this parasitoid (N. Mills, personal observation), climatic tolerance is likely to be an important driver of its current distribution. We thus used the spatially explicit climatic data available from the WorldClim data set [32] at 5-arcminute resolution, which comprises 19 bioclimatic variables representing annual trends, seasonality and extremes of temperature and precipitation. These variables are collected and interpolated from globally distributed weather stations and are averaged over a period ranging from ∼1950–2000. In general, temperature and precipitation have been used extensively to model the distribution of insects [33]–[36], and the WorldClim data set has been a central source of climate data for modeling species distributions [37], [38].

We first attempted to minimize model over-fitting by calculating Pearson's correlation coefficient (*r*) between each pair of variables for 1,000 randomly selected points from throughout the geographical extent selected for bioclimatic modeling. Climate data for each point were extracted using DIVA-GIS v5.4 (http://www.diva-gis.org). Variables with *r*>0.80 were considered as highly correlated and we selectively removed one variable from each of these pairs. Whenever possible, we retained variables that represent climatic seasonality or extremes rather than annual averages of precipitation and temperature, as they seem more likely to influence the range limits of a seasonal species like *A. transcaspicus*. The final model included 10 variables: BIO2 – mean diurnal temperature range [(mean of monthly (maximum – minimum temperature)]; BIO3 – isothermality [(mean monthly temperature range/annual temperature range)×100]; BIO4 - temperature seasonality (SD×100); BIO5 – maximum temperature of the warmest month; BIO6 – minimum temperature of the coldest month; BIO8 – mean temperature of the wettest quarter; BIO9 – mean temperature of the driest quarter; BIO13 – precipitation of the wettest month; BIO14 – precipitation of the driest month; and BIO15 – precipitation seasonality (coefficient of variation). In comparison to the ENM from the complete set of variables (not shown) this reduced set did not alter the results to a great extent, although it generated an ENM with a slightly broader distribution. Thus the model presented here is likely to have a lower rate of falsely omitted cells and should be a more conservative distribution against which to evaluate barriers to gene flow in *A. transcaspicus* based on predictions of climatic unsuitability.

We used logistic output from Maxent which generates a probability of presence (range 0–1) to each grid cell [39]. During initial runs we randomly selected 70% of sampling localities as training data and 30% as test data [31]. Thus, from the 81 records, we had 45 training points and 19 test points, after excluding multiple occurrences per grid cell. We ran Maxent 10 times with default parameters to evaluate performance across runs. For each we used the area under the receiver operating characteristic curve (AUC) to evaluate model performance for both training and test data. The AUC statistic is a threshold-independent measure of model performance; theoretically, an AUC score of 1.0 indicates optimal performance, while AUC = 0.5 indicates a model that predicted occurrences no better than at random [40]. We also used a threshold dependent one-tailed binomial omission test to test the hypothesis that test points are predicted no better than at random for each of 11 thresholds implemented in Maxent v3.1. Following evaluation, we performed a single final run of the model using all non-duplicate sample coordinates, following [31]. This final full model is presented below (referred to as the ‘full ENM’) as a best estimate for the potential niche-based distribution of *A. transcaspicus* in the Mediterranean.

For visualization of ENM predictions, unsuitable habitat was defined as grid cells with a logistic probability less than the lowest value for any sampled locality (after exclusion of the Turkish locality as an outlier, see below). The remaining grid cells are presented continuously from light to dark shades representing the lowest to highest probability of occurrence, respectively. Finally, the importance of each variable to the final model was analyzed using the jackknife procedure implemented in Maxent, where separate models are analyzed that include each variable alone; the resulting effect on training gain can be viewed as a measure of the information contained by the particular variable.

#### Among-Cluster Climatic Differences

To further examine whether different genetic clusters occupied distinct ecological niche space, in addition to the ‘full ENM,’ we created two ‘reduced ENMs’ using only localities from either *K*1 or from *K*3 following the procedures above. *K*2 was not considered due to the small number of observations.

We also investigated how the climate experienced by *A. transcaspicus* differed among the three genetic clusters. We extracted values for the 10 bioclimatic variables used in the full niche model for each unique coordinate belonging to *K*1, *K*2, and *K*3 (making the assumption that ungenotyped localities in the vicinity of genotyped localities belonged to the same genetic cluster). We performed a principal components analysis (PCA) on correlations between variables using JMP (SAS Institute) followed by analysis of variance (ANOVA) among clusters on each of the first three principal components (PC1, PC2, PC3). We also tested the significance of differences between *K*1 and *K*3 localities separately for each climate variable using Students *t*-tests (once again *K*2 was excluded because of sample size). Bonferroni correction was applied so that α = 0.005 for 10 comparisons.

## Results

### Isolation with Migration Model

We attempted to compare analyses for all three pairwise population cluster comparisons, though we were unable to achieve MCMC convergence for those including *K*2, so only the *K*1−*K*3 comparison is presented. Multiple runs of IMa converged on very similar posterior probability distributions over multiple short analyses (not shown), and below we present a final analysis consisting of 5×10^5^ sampled genealogies (Table 1). All posterior distributions rose and fell from zero, with a clearly defined unimodal peak. Estimates of θ for *K*1 and *K*3 were very similar and were both much smaller than the θ_A_. The migration estimates were small in both directions, and the 90% HPDs for these parameters overlapped the smallest histogram bin (0.005). The highest posterior probabilities for migration were 0.305 and 0.585 for *m*_K1_ and *m*_K3_, respectively. The effective rate at which individuals enter a population per generation (*Nm*; assuming equal sex ratios) was found by multiplying *m*_K1_ and *m*_K2_ by the highest posterior probability estimates of θ_K1_ and θ_K3_, respectively and then dividing the resulting values by three. This results in mean estimates of 0.036 effective migrants into *K*1 and 0.078 into *K*3 per generation. The likelihood ratio tests showed that the full *IM* model was not significantly better than the equal migration model, which in turn was not significantly better than the zero migration model (Table 1). Neither of the shorter preliminary runs was significant for either test (not shown). Together these results demonstrate that considering gene flow between *K*1 and *K*3 is not necessary to explain patterns of genetic variation in these populations, and that a pure isolation model is sufficient.

**Table 1:** Estimates of demographic parameters from IMa analysis with results of model comparisons.

	Maximum PP^a^	90% HPD^b^
θ_K1_	0.354	0.149–0.697
θ_K3_	0.400	0.178–0.790
θ_A_	61.003	19.877–217.280
*m*_K1_	0.305	0.005–1.515
*m*_K3_	0.585	0.005–2.295
Τ	0.202	0.058–0.462

	log(*P*)	−2LLR
Full Model	−2.978	-
*m*_K1_ = *m*_K3_	−4.757	3.558^c^ (*P* = 0.06)
*m*_K1_ = *m*_K3_ = 0	−5.406	1.298^d^ (*P* = 0.13)

a Maximum posterior probability estimate.

b 90% highest probability marginal-density.

c To determine whether the full model was significantly better than the equal migration model, the likelihood ratio (−2LLR) statistic was tested using a χ^2^ distribution with 1 d.f.

d To determine whether the equal migration model was better than the no migration model the −2LLR statistic was tested using a mixed distribution of ½χ^2^_1_+½ χ^2^_0_.

### Ecological niche model

Maxent appeared to perform well for the full ENM, with average training and test AUC values of 0.961±0.004 SD and 0.944±0.015 SD, respectively, across 10 replicate runs. Threshold-based tests also suggest that the model predicted test localities significantly better than at random, with binomial probabilities of *P*<5×10^−4^ for all omission thresholds examined. In the model generated using all localities as training data, the only locality omitted from the predicted distribution was from Kahramanmaras, Turkey. The climatic variables that contributed most to the model are summarized in Table 2 indicating that the minimum temperature of the coldest month and temperature seasonality contributed most to the full ENM.

**Table 2:** Analysis of bioclimatic variable importance for the three ENMs.

	Full ENM		*K*1-only ENM		*K*3-only ENM	
	Percent contribution^a^	Jackknife TG^b^	Percent contribution^a^	Jackknife TG^b^	Percent contribution^a^	Jackknife TG^b^
BIO6-Min. temp. coldest month	34.84	1.111	19.21	1.043	51.98	1.836
BIO4-Temp. seasonality	22.19	1.254	34.07	1.533	1.39	1.257
BIO2-Mean monthly temp. range	17.06	0.639	15.64	0.733	3.64	0.833
BIO13-Precip. wettest month	7.81	0.674	1.34	0.408	35.30	1.193
BIO14-Precip. driest month	5.80	0.290	9.45	0.300	0.37	0.300
BIO9-Mean temp. driest quarter	5.67	0.877	5.60	0.939	1.99	0.705
BIO3-Isothermality	3.24	0.339	13.14	0.838	0.14	0.236
BIO15-Precip. seasonality	2.07	0.263	0.04	0.243	0.00	0.419
BIO8-Mean temp. wettest quarter	1.17	0.384	1.38	0.333	3.11	0.357
BIO5-Max. temp. warmest month	0.16	0.668	0.14	0.719	2.08	0.420

a Assessment of relative contributions of environmental variables to total gain in the Maxent model.

b Jackknife tests of regularized training gain (TG) for models run with each variable alone.

**Figure 2:**
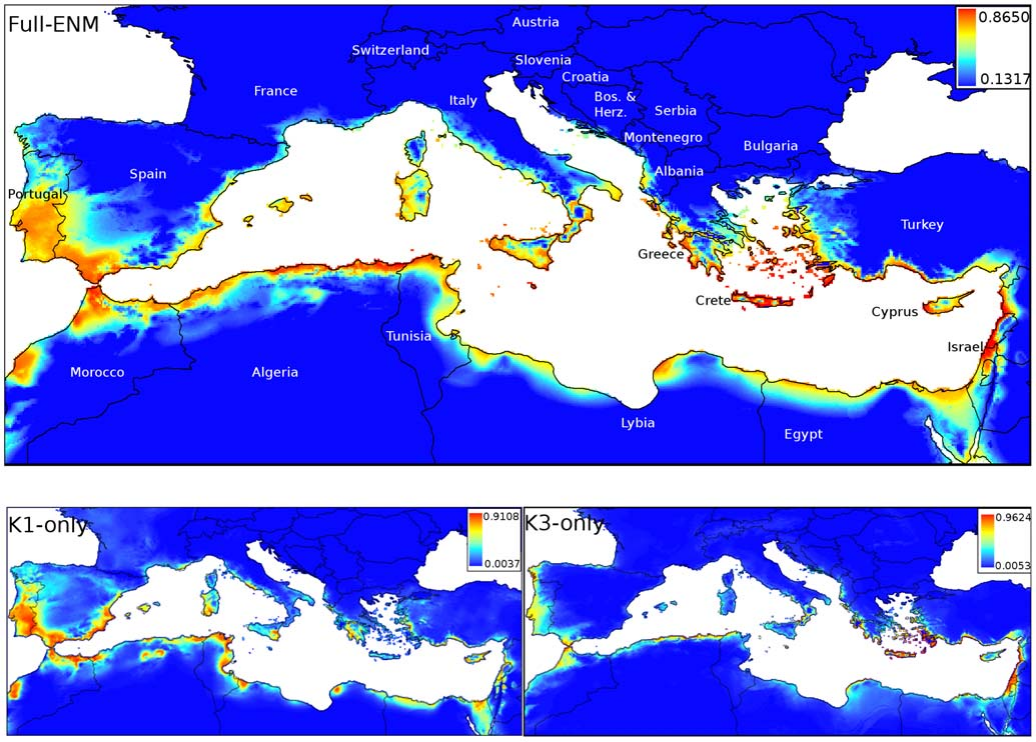
Spatial distribution predictions generated for *A. transcaspicus* by Maxent using all localities (Full ENM), only parasitoids from *K*1 localities (*K*1-only ENM), and only parasitoids from *K*3 localities (*K*3-only ENM). Levels of shading represent continuous logistic probabilities of bioclimatic suitability, corresponding to highest suitability (red) to unsuitable (blue) habitat. Unsuitability thresholds were set at the lowest logistic probability of occurrence for any locality included as a training point in the given ENM.

The spatial prediction generated for the full ENM was largely congruent with our prior expectations for the distribution of *A. transcaspicus* in the Mediterranean (Figure 2). Grid cells with the highest occurrence probabilities were distributed in coastal areas in the southern-most parts of Europe and northern-most parts of Africa, as well as on most Mediterranean islands, and suitability gradually declined with increasing distance from the coast. Localities in the region occupied by the *K*1 cluster were connected by large, continuous areas of high suitability, including the Iberian Peninsula, Morocco, Algeria, and Tunisia, as well as coastal areas to the east of Tunisia from which we had no samples. The only break in the predicted distribution within the *K*1 region was caused by the Straits of Gibraltar, a distance of ∼15 km over water.

Predicted habitat suitability between *K*1 and *K*2 was not continuous, however. While much of Spain was predicted to be at least moderately suitable for *A. transcaspicus*, habitat suitability in the *K*1 region declined strongly along the coast in northern Spain at about 40–41°N latitude, with only a narrow strip of habitat with low probability of occurrence along the coast of France and a lack of suitable habitat for about 75–100 km along the Italian coast to the east of France. This prediction of low suitability is supported by our failure to collect *A. transcaspicus* in France and northern Italy, despite the presence of their hosts (e.g., Figure 1).

The most striking feature of the full ENM, however, is the substantial area of unsuitable habitat predicted to occur between the *K*1+*K*2 and the *K*3 regions. Even coastal areas fall outside of the predicted distribution in this intervening region surrounding the Adriatic Sea, including northern Italy, Slovenia, Croatia, Montenegro, as well as inland portions of Albania and northern Greece. Greece, however, did show relatively high connectivity with other *K*3 countries including Turkey, Israel, and Egypt, either over land (e.g. the Turkish coast) or over islands of high predicted suitability in the Aegean Sea. Furthermore, a distinct break in the ENM prediction is apparent in northern Africa (Libya), which could help explain the genetic discontinuity between *K*1 and *K*3 in this part of the *A. transcaspicus* range.

### Ecological similarity among genetic clusters

**Figure 3:**
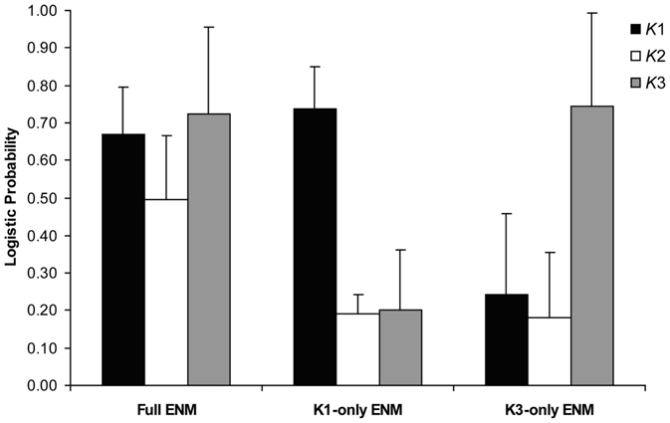
Average logistic probability of presence (±1 SD) for localities in *K*1 (*n* = 30), *K*2 (*n* = 5), and *K*3 (*n* = 28) population clusters in each of the examined ENMs.

The reduced ENMs developed using localities from either *K*1 (average training and test AUC values of 0.994±0.001 SD and 0.992±0.002 SD, respectively) or *K*3 (average training and test AUC values of 0.981±0.004 SD and 0.982±0.018 SD, respectively) alone also performed well in predicting the range-wide distribution of *A. transcaspicus* (Figure 2). Each of these predictions is broadly similar in scope to the full ENM, particularly the *K*1-only ENM. As in the full model, both reduced models predicted a coastal restriction for *A. transcaspicus* and also identified breaks in bioclimatic suitability found along the central Libyan coast and along the northern coast of the Adriatic Sea. Overall this suggests that there is some level of niche similarity between the genetic clusters, though there are several important differences. For the *K*1-only ENM none of the localities in *K*1 or *K*2 were omitted completely from the predicted distribution. Most of the *K*3 localities were also predicted successfully, with the exception of 5 localities in Israel, the Turkey locality, and a single locality from Pilion-Magnisia, Greece that was georeferenced from [30]. The omissions from the *K*3-only model were more pronounced, with regions containing the Turkey and Pilion-Magnisia [30] localities (*K*3), the three Italian localities (*K*2), and the southernmost Tunisian and all eastern Spanish localities (*K*1) classified as unsuitable. Comparison of logistic probabilities of occurrence among clusters and models shows clear declines for localities not included in reduced models relative to the full model (Figure 3). While, on average all sampled localities were predicted with high probability in the full ENM, in the *K*1-only ENM, the *K*2 and *K*3 localities were dramatically reduced, and likewise, for the *K*3-only ENM the *K*1 and *K*2 localities had a low probability of occurrence (Figure 3). This suggests that localities in each cluster contain unique bioclimatic niche information and thus a model generated using data from only a single cluster does a poor job of predicting the detailed geographic distributions of parasitoids in the other clusters, even though, at least qualitatively, the *K*1-only ENM does a fairly good job of reproducing the distribution map from the full ENM.

**Figure 4:**
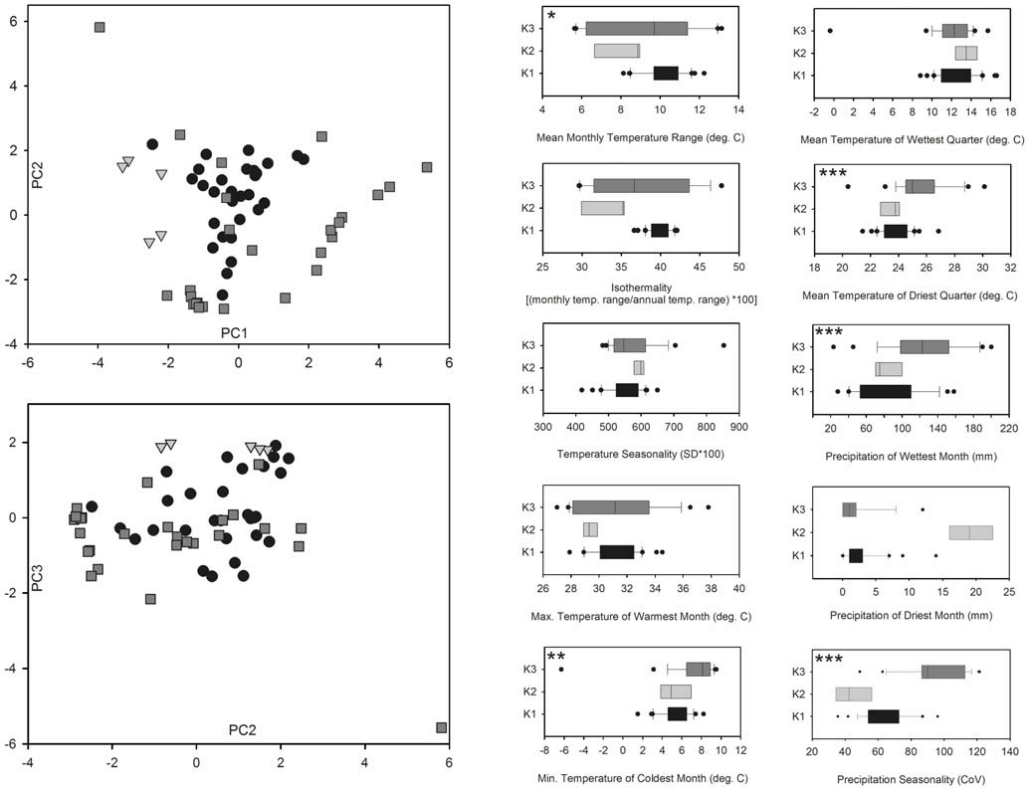
Comparison of bioclimatic variables experienced at localities sampled within each *A. transcaspicus* genetic cluster (see Figure 1). A) Separation of clusters (*K*1: black circles, *K*2: light gray triangles; *K*3: dark gray squares) along the first (PC1) and second (PC2) or second and third (PC3) principal components derived from an analysis performed on variables shown in (B). B) Box plot comparisons among *K*1−*K*3 for individual climatic variables used for Maxent predictions. Significance of differences was tested by *t* tests only between *K*1 and *K*3, with *K*2 values shown only for comparison. * = *P*<0.05; ** = *P*<0.01; *** = *P*<0.001 (see Results for details on significant tests).

The first three axes of the PCA on bioclimatic variables extracted from sampling localities (explaining 35%, 32%, and 15% of the variance respectively) show that the three Mediterranean *A. transcaspicus* clusters broadly overlap in multidimensional niche space (Figure 4). However, ANOVAs were significant among parasitoid clusters for all three principal components (PC1: *F*_2. 60_ = 8.49, *P*<0.001; PC2: *F*_2. 60_ = 4.64, *P*<0.05; PC3: *F*_2. 60_ = 12.11, *P*<0.001 ), indicating some differences in the bioclimatic niche space occupied by parasitoids in each region, as suggested by locality omissions in the reduced ENMs. When compared individually between *K*1 (*n* = 30) and *K*3 (*n* = 28) localities, five of the 10 bioclimatic variables showed significant differences (Figure 4): mean monthly temperature range (BIO2; *t*_56_ = 2.14, *P* = 0.036, although this test is not significant following Bonferroni correction to α = 0.005); minimum temperature of the coldest month (BIO6; *t*_56_ = 2.96, *P* = 0.005); mean temperature of the driest quarter (BIO9; *t*_56_ = 3.86, *P*<0.001); precipitation of the wettest month (BIO13; *t*_56_ = 4.21, *P*<0.001); and precipitation seasonality (BIO15; *t*_56_ = 6.81, *P*<0.001). As in the ENMs, the Turkish locality was a noticeable outlier in most plots, again suggesting that this point falls outside the bioclimatic zone typical for *A. transcaspicus* in the Mediterranean. However, repeating the PCA and *t* tests on bioclimatic variables after exclusion of this locality did not change any patterns of significance (not shown).

## Discussion

The power of integrating genetic, ecological, and distributional data when studying the process of diversification is being increasingly recognized, with such approaches providing insights into the mechanisms of evolutionary divergence (e.g. allopatry versus sympatry; drift versus natural selection and adaptation) and also aiding practical taxonomic decisions [3], [7], [9], [41]. In the Mediterranean region, the parasitoid *A. transcaspicus* is subdivided into three major population clusters that encompass the Iberian Peninsula and the Maghreb region of northern Africa (*K*1), Italy (*K*2), and the eastern Mediterranean (*K*3; Figure 1) [19]. Given the high degree of genetic structure among these clusters [19] and experimental data suggesting the possibility of some pre-mating reproductive barriers [21] (Lozier & Mills in prep.), we wanted to better understand how populations of *A. transcaspicus* are connected by gene flow. In the present study we quantified gene flow using a coalescent-based analysis under a model of isolation with migration and, using an ecological niche modeling approach, tested whether predicted distributions support patterns of strong population structure in the Mediterranean. Our results show that the spatial distribution of *A. transcaspicus* is likely limited, at least in part, by climatic tolerances. The prediction of three main geographic regions of high environmental suitability separated by regions of poor suitability is a plausible explanation for the nearly complete genetic isolation of the previously identified population clusters, and suggests that environmental variables may provide the gene flow barriers necessary in the early stages of allopatric speciation.

### Distinguishing Isolation from Migration

Previous estimates of high genetic differentiation [19] were supported by our analyses of gene flow using the coalescent *IM* model. Modal values of the posterior probability distributions for the two migration parameters were consistent with very low levels of ongoing post-divergence migration between western (*K*1) and eastern (*K*3) Mediterranean populations, well below that often considered necessary for ecological or evolutionary trajectories to remain correlated [42]. Furthermore, the *IM* models including symmetrical or asymmetrical migration parameters did not have significantly greater likelihood than the nested model excluding gene flow, indicating that contemporary migration is not necessary to explain the observed patterns of genetic variation (Table 1). While we cannot completely rule out low levels of ongoing migration with only six loci, given the present data we conclude that gene flow between these two *A. transcaspicus* populations is likely to be negligible and that they have remained effectively reproductively isolated since divergence. This suggests that mtDNA haplotypes shared between the two populations are likely the result of incomplete lineage sorting [13] rather than ongoing dispersal.

#### Effects of Unsampled Populations on Migration Inferences

It is unfortunate that models comparing *K*2 were not possible given the failure of MCMC convergence in IMa, likely due to the more limited sample sizes for Italian populations. It thus becomes important to consider how the absence of IMa results for *K*2 might influence our interpretation of results for *K*1 and *K*2. One possibility is that the *K*2 population, due to its intermediate geographic location (Figure 1), might act as a stepping-stone for dispersal between *K*1 and *K*3. Using the five microsatellite loci evaluated in the present study, an estimate of *F*_ST_ is, in fact, smaller between *K*1 and *K*2 (*F*_ST_ = 0.487) than between *K*1 and *K*3 (*F*_ST_ = 0.657), suggesting that there may be some slightly higher level of gene flow between western Mediterranean and Italian *A. transcaspicus* populations. Although this level of divergence is still very high for microsatellite data. The *F*_ST_ observed for *K*2 and *K*3 (*F*_ST_ = 0.601), however, is nearly as high as for *K*1 and *K*3, suggesting that genetic isolation for Italian and eastern Mediterranean populations is likely to be as strong as that observed here for western and eastern Mediterranean populations. Furthermore, any indirect gene flow between the eastern and western Mediterranean populations through Italy, or some other unsampled population, should still have been inferred as *direct* post-divergence migration in the *IM* model [43], [44] rather than no migration. We thus expect that the high degree of reproductive isolation observed between the western and eastern Mediterranean *A. transcaspicus* populations is unlikely to be affected by the absence of results for *K*2. We anticipate that additional sampling of parasitoids from Italy will reveal that *K*2 does not act as a major stepping-stone population in the Mediterranean, although the degree to which this population is connected by gene flow to either *K*1 or *K*3 remains to be assessed.

### Ecological Niche Models

Parasitoids belonging to the *K*1 genetic cluster are broadly distributed in Spain and the Maghreb region of Africa (represented here by Morocco and Tunisia). These regions were well connected in the ENM, with only the Straits of Gibraltar as a potential barrier to dispersal. While previous studies have identified the Straits of Gibraltar as a strong phylogeographic barrier between Iberia and Africa for some organisms [45], [46], movement across the straits is known for several insect species [47], [48]. Such a narrow stretch of water may not be an effective dispersal barrier for a flying insect like *A. transcaspicus*. At the northern border of the *K*1 cluster in Europe the full and *K*1-only ENMs predicted that habitat suitability declines between northern Spain into France, with only a small strip of coastal habitat predicted as marginally suitable for *A. transcaspicus*, corroborating our failure to collect *A. transcaspicus* in northern Spain and southern France. The models also suggest that parasitoids in the *K*2 cluster are likely to be restricted largely to southern and eastern coastal Italy. Again, this is consistent with absences during field observations (Figure 1). Finally, the ENMs predicted two major over-land breaks in environmental suitability—one between Italy and Greece and another along the central Libyan coast—that correspond strongly with the genetic distinctness of *A. transcaspicus* in the eastern Mediterranean (*K*3). Within the eastern Mediterranean, connectivity of predicted suitable habitat was high, both along the mainland coast as well as for islands groups. While we only had samples for the islands of Crete and Cyprus, our results suggest that *A. transcaspicus* is also likely to be present on many other islands in the region.

In summary, the ENMs show that *A. transcaspicus* is likely to be restricted largely to the coastal Mediterranean, with climatic suitability generally declining inland. This supports the prediction that *A. transcaspicus* prefers “Mediterranean” environments to other habitat types found in the region [30] (N Mills personal observation). Together with results from previous analyses [19], these data suggest that regions of environmental unsuitability may be effective barriers to dispersal for *A. transcaspicus*, even more so than short stretches of water, and may explain the patterns of genetic structure present in the Mediterranean.

One potential problem with identifying breaks in species distributions using ENMs is the difficulty in determining if such breaks are due to true environmental unsuitability or false omissions due to insufficient sampling effort. This is most problematic for the *K2–K3* gap in the eastern Balkans, for which we have no observations of *A. transcaspicus*. Although a recent review of aphid parasitoids of southeastern Europe reported no evidence of *A. transcaspicus* from these countries [30], additional sampling to verify its absence in this region would be ideal.

Another anomaly that suggests model under-prediction is the omission of the Turkish locality of Kahramanmaras from all three ENMs. However, because this locality was an obvious outlier for most environmental variables, we favor an alternative explanation. Based on the label data included with the Turkish *A. transcaspicus* specimens (supplied by P. Starý), this locality was georeferenced to the city of Kahramanmaras, but we suspect that this may not be an accurate representation of the site of collection. The Turkish *province* Kahramanmaras extends well to the south of the city into an area predicted to be highly suitable for *A. transcaspicus* in our ENMs, and may represent the true source of these samples. This single locality did not affect our results, but does highlight a potential concern for generating ENMs if accurate georeferencing is not available for many data points [but see 49].

Based on our own collecting experience, there is even some reason to believe that the ENM for *A. transcaspicus* may actually over-predict the true geographic range of this species. This is a general concern when evaluating ENM predictions; these methods generate *potential* distributions based only on the environmental variables provided by the user, and will not accurately predict aspects of a distribution that are influenced by biotic or abiotic factors not included in the model. For example, ENMs will over-predict a species' range if climatic conditions in an area are suitable, but there is some external factor that prevents colonization, such as the presence of a competitor, absence of prey, or physical barrier such as a mountain range. Beyond the climatic conditions included in our ENMs, the distribution of *A. transcaspicus* will be affected by the distribution and abundance of *Hyalopterus* aphids, which in turn will be affected by the distribution of suitable primary and secondary host plants. Such factors might explain the high suitability predicted for areas along the Atlantic coast of Morocco despite our failure to detect *A. transcaspicus* in this region in 2006, as *Hyalopterus* aphids were also rare. However, in other areas, such as southern France, *Hyalopterus* are abundant, and we have never observed *A. transcaspicus* in this region. *Hyalopterus* species are, in fact, broadly distributed in Europe and Africa [23] and in many parts of the world where they are invasive [50], suggesting that the presence of hosts is not the most important factor limiting the distribution of *A. transcaspicus*. Indeed, localities where *Hyalopterus* was collected but *A. transcaspicus* was absent generally had low probability of occurrence in the ENM (Figures 1, 2), suggesting that the climatic variables included in the model predict unsuitable habit with good reliability.

#### Niche Similarity Among Populations

The results of our ENM analyses suggest that the niche space occupied by parasitoids belonging to different populations is similar, but certainly not identical. The ENMs generated using only a subset of localities from either the *K*1 or *K*3 regions resulted in range predictions with boundaries that broadly agreed with the full model, suggesting that parasitoids in each population may experience many of the same environmental conditions. All three ENMs also showed gaps in habitat suitability in the same geographic regions, suggesting that these breaks are climatically unsuitable for parasitoids from each population. These observations are consistent with niche conservatism driving genetic isolation in *A. transcaspicus*, where diverging lineages possess adaptations to a recently shared environment and will tend to occur in ecologically similar areas separated by unsuitable habitat [5]. However notable differences in the reduced ENMs, multivariate analysis, and direct comparisons of climatic variables also suggest a potential for niche evolution within the areas inhabited by the different genetically distinct populations, where natural selection causes niche parameters to diverge in association with lineages [51]. Bioclimatic variables that significantly differed between the clusters *K*1 and *K*3 indicated that, on average, *K*1 localities appear to experience smaller seasonal precipitation fluctuations, colder winter temperatures, cooler temperatures in dry summer months, and less precipitation in the wettest winter month. While we did not statistically compare variables for *K*2 because of the small number of localities available, the trends apparent in Figure 4 suggest that in some cases southern Italy shares climatic characteristics with either *K*1 or *K*3 localities, but in others appears distinct (e.g. greater precipitation in the driest month, less precipitation seasonality).

The potential importance of these climatic differences for niche evolution in *A. transcaspicus* populations merits further research. Biological control practitioners have long recognized the need to consider climate when selecting natural enemy strains for pest management because parasitoid species often appear to exhibit adaptations to local climate in different geographic regions [52]–[54]. For example, overwintering success of aphid parasitoids can be influenced by their level of cold tolerance [55], [56], and while there have been no studies of fungal infection of overwintering parasitoid mummies, infections of overwintering aphidophagous predators can be much greater under conditions of higher precipitation [57]. At the opposite extreme, in summer, parasitoids have a maximum temperature tolerance for survival which can also vary according to geographic region [58], [59]. Given the ease of rearing *A.transcaspicus* in the laboratory, it would be relatively straightforward to conduct experiments to test the effects of different ambient temperatures and moisture levels on colonies collected from different geographic regions. Studies on the climatic limitations for *A. transcaspicus* would also act as useful validation for the ENM presented here.

### Synthesis

Given results from the *IM* analysis and ENMs, we can begin to speculate on the recent evolutionary history of *A. transcaspicus* in the Mediterranean. We were clearly able to identify a lack of contemporary gene flow between environmentally isolated populations in the eastern and western Mediterranean, although our ENM only applies to a typical 20^th^ century climate, and it is more difficult to make inferences regarding demographic processes that occurred prior to this period or how changes in climate may affect future patterns of dispersal and gene flow. And while our genetic results suggest that populations have been isolated since they diverged, estimating the divergence time itself is problematic with the present data. Problems in conversion of coalescent parameters to demographic values can arise, for example, from unpredictable scaling associated with intra-population substructure (although within-population structure is minimal for *A. transcaspicus* [19]) or due to uncertainty in mutation rates (μ) for molecular markers. However, obtaining a broad idea of divergence times can still be valuable for developing hypotheses about demographic history. By applying a naïve estimate of the geometric mean μ for the six markers employed in our IMa runs based on the geometric mean of 10^−4^ mutations per generation for microsatellite loci [60] and 10^−8^ substitutions per site per year for *COI* [61], we obtain a geometric mean μ = 4.04×10^−5^ per gene per generation, assuming 10 generations per year (estimation based on laboratory observations and probable activity period in the field). The use of this rate leads to a divergence time estimate of ∼5,000 generations, or ∼500 (90% HPD: 143–1,145) years. However, microsatellite loci are known to have much slower rates of evolution for some insects, which would make true divergence times much older. For instance the use of a lower microsatellite mutation rate such as that observed in *Drosophila melanogaster* (5×10^−6^ per generation) [62] leads to a divergence time of 6,076 (90% HPD: 1,745–13,896) years. In reality mutation rates will differ across microsatellite loci [60] so the accuracy of either estimate cannot really be assessed, though population divergence at some point within the last 15–20,000 years seems a reasonable upper bound.

Given such uncertainty in the divergence time, there are a number of possible hypotheses for the origins of genetic structure in *A. transcaspicus*, although resolution among these will require further study. One possibility is that the present distribution of isolated populations originated during the last glacial maximum in Europe. Climatic oscillations have led to repeated expansion and contraction of Mediterranean species, with allopatric isolation in Pleistocene refugia, in particular, having been implicated in shaping the present genetic structure of much of the European biota [63]–[65]. Interestingly, our ENM results suggest that the distribution of *A. transcaspicus* today differs little from that which might be expected for Pleistocene refugia of many European species [e.g. see Figure 1 of Ref. 65]. If the observed genetic differentiation is indeed indicative of Pleistocene isolation, why might *A. transcaspicus* not have expanded further northward into Europe as suitable habitat became available? It is possible that Pleistocene populations were distributed as they are today, but remained trapped in their refugia, perhaps due in part to an inability to cross the east-west oriented mountain ranges dominant in Mediterranean refuges. This hypothesis would suggest that *A. transcaspicus* would have had to evolve rapidly in response to changing conditions, and could account for the somewhat different climatic niches occupied by the different genetic population clusters. Alternatively, *A. transcaspicus* may have been isolated in much smaller refugia with appropriate climatic conditions, perhaps at the southern limits of European peninsulas or in northern-most Africa, and subsequently expanded into the present distribution as favorable conditions spread. This latter hypothesis makes more sense in light of the low variation observed at *COI* (seven total haplotypes dominated by two sequences present in 94% of individuals) and microsatellite loci (average expected heterozygosity across all localities of 0.37), as well as the absence of isolation by distance within population clusters [19], a pattern typical of non-equilibrium demographic histories [66].

A second scenario could involve even more recent processes consistent with dates associated with the faster (10^−4^ per generation) microsatellite mutation rate. For example, it is plausible that *A. transcaspicus* spent the last glaciation completely outside of the Mediterranean (possibly in Asia, the suspected origin of the sibling species *A. colemani*) [27], and was introduced rapidly through the Mediterranean by human action associated with the spread of cultivated *Prunus* and their associated *Hyalopterus* aphids [67], [68]. Climatically unsuitable regions located between those currently occupied by the three contemporary Mediterranean population clusters would have reduced the likelihood of establishment in these areas, thereby preventing gene flow and allowing for the development of genetic differentiation. Recent introduction followed by rapid isolation due to climatic barriers is also consistent with the low within-population genetic variation and lack of isolation by distance discussed above. Inter-population cluster differentiation would be exacerbated on this recent time-scale if, as the IMa result suggests, founding populations were small relative to the ancestral population, allowing drift to more effectively change allele frequencies in the absence of gene flow (although we note that estimates of θ_A_ can be inflated in an *IM* model by the presence of ‘unsampled’ contemporary populations, such as *K*2 and Middle Eastern populations not considered here). This hypothesis is particularly intriguing in light of partial reductions in mating success observed in reciprocal crosses between *A. transcaspicus* collected in Spain and Israel [21] (Lozier & Mills, in prep.), and would indicate extremely rapid evolution of pre-mating reproductive isolation behaviors. This might not be particularly surprising for an insect with many generations per year, but would still be an interesting example of rapid divergence in response to recently imposed allopatric isolation. Paleodistributional modeling could be a useful tool for determining where *A. transcaspicus* could have been historically distributed given its current climatic tolerances [69], [70], and thus for distinguishing these different hypotheses. This approach may not be suitable, however, if recent niche evolution has occurred because conditions favorable to *A. transcaspicus* today may not match those experienced by historical populations. However, a better understanding of how *Hyalopterus* and their *Prunus* host plants were distributed during the Pleistocene may provide important clues as to where *A. transcaspicus* could have survived this period.

In conclusion, our results highlight the power of integrating multiple approaches for understanding patterns of divergence in closely related populations. By incorporating climatic niche-based models of geographic distribution and statistical analysis of isolation versus migration in situations where reciprocal monophyly is unlikely, researchers can not only more effectively detect cryptic taxonomic diversity but can better understand the mechanisms that generate this diversity. Our results suggest that *A. transcaspicus* may warrant further attention by Aphidiine systematists to determine if any morphological differences correlate with patterns of geographic genetic structure. While we have found evidence for environmentally mediated geographic isolation in contemporary populations, questions persist as to the historical events that may have resulted in the current distribution of *A. transcaspicus*. More data from *A. transcaspicus* and its close relatives, together with better estimates of intra-specific substitution rates and more complex population genetic models, will allow us to further resolve divergence times and colonization processes for these Mediterranean populations.

## Supporting Information

Table S1 Supplementary coordinate data. Coordinates for presence localities used in the ecological niche models
(0.12 MB DOC)

## References

[1] Palsbøll PJ, Bérubé M, Allendorf FW (2007). Identification of management units using population genetic data.. Trends Ecol Evolut.

[2] Barraclough TG, Vogler AP (2000). Detecting the geographical pattern of speciation from species-level phylogenies.. Am Nat.

[3] Bond J, Stockman A (2008). An integrative method for delimiting cohesion species: finding the population-species interface in a group of Californian trapdoor spiders with extreme genetic divergence and geographic structuring.. Syst Biol.

[4] Graham CH, Ron SR, Santos JC, Schneider CJ, Moritz C (2004). Integrating phylogenetics and environmental niche models to explore speciation mechanisms in dendrobatid frogs.. Evolution.

[5] Wiens JJ, Graham CH (2005). Niche conservatism: integrating evolution, ecology, and conservation biology.. Annu Rev Ecol Evol Systemat.

[6] Ruegg KC, Hijmans RJ, Moritz C (2006). Climate change and the origin of migratory pathways in the Swainson's thrush, *Catharus ustulatus*.. J Biogeogr.

[7] Rissler LJ, Hijmans RJ, Graham CH, Moritz C, Wake DB (2006). Phylogeographic lineages and species comparisons in conservation analyses: a case study of California herpetofauna.. Am Nat.

[8] Schluter D (2001). Ecology and the origin of species.. Trends Ecol Evolut.

[9] Raxworthy CJ, Ingram CM, Rabibisoa N, Pearson RG (2007). Applications of Ecological Niche Modeling for Species Delimitation: A Review and Empirical Evaluation Using Day Geckos (*Phelsuma*) from Madagascar.. Syst Biol.

[10] Kozak KH, Graham CH, Wiens JJ (2008). Integrating GIS-based environmental data into evolutionary biology.. Trends Ecol Evolut.

[11] Kozak KH, Wiens JJ (2006). Does niche conservatism promote speciation? A case study in North American salamanders.. Evolution.

[12] Rissler LJ, Apodaca JJ (2007). Adding more ecology into species delimitation: ecological niche models and phylogeography help define cryptic species in the black salamander (*Aneides flavipunctatus*).. Syst Biol.

[13] Maddison WP (1997). Gene trees in species trees.. Syst Biol.

[14] Whitlock MC, McCauley DE (1999). Indirect measures of gene flow and migration: F_ST_≠1/(4 Nm+1).. Heredity.

[15] Hudson RR (1991). Gene genealogies and the coalescent process.. Ox Surv Evol Biol.

[16] Hey J, Nielsen R (2004). Multilocus methods for estimating population sizes, migration rates and divergence time, with applications to the divergence of *Drosophila pseudoobscura* and *D. persimilis*.. Genetics.

[17] Hey J, Nielsen R (2007). Integration within the Felsenstein equation for improved Markov chain Monte Carlo methods in population genetics.. Proc Natl Acad Sci USA.

[18] Niemiller ML, Fitzpatrick BM, Miller BY (2008). Recent divergence with gene flow in Tennessee cave salamanders (Plethodontidae: Gyrinophilus) inferred from gene genealogies.. Mol Ecol.

[19] Lozier JD, Roderick GK, Mills NJ (2008). Evolutionarily significant units in natural enemies: Identifying regional populations of *Aphidius transcaspicus* (Hymenoptera : Braconidae) for use in biological control of mealy plum aphid.. Biol Contr.

[20] Excoffier L, Smouse PE, Quattro JM (1992). Analysis of molecular variance inferred from metric distances among DNA haplotypes - application to human mitochondrial-DNA restriction data.. Genetics.

[21] Lozier J (2007). Population genetic and ecological approaches for improving biological control of mealy plum aphid, *Hyalopterus pruni*, in California..

[22] Romani R, Rosi MC, Isidoro N, Bin F (2008). The role of the antennae during courtship behavior in the parasitic wasp *Trichopria drosophilae*.. J Exp Biol.

[23] Blackman RL, Eastop VF (2000). Aphids on the World's Crops, 2^nd^ Edition.

[24] Lozier JD, Mills NJ, Roderick GK (2006). Di- and trinucleotide repeat microsatellites for the parasitoid wasp, *Aphidius transcaspicus*.. Mol Ecol Notes.

[25] R Development Core Team (2008). R: A language and environment for statistical computing.

[26] Bolker B (2007). emdbook: Ecological models and data. R package version 1.1.1.. http://www.zoo.ufl.edu/bolker/emdbook.

[27] Starý P (1975). *Aphidius colemani* Viereck: its taxonomy, distribution and host range (Hymenoptera: Aphidiidae).. Acta Entomol Bohemoslov.

[28] Mescheloff M, Rosen D (1990). Biosystematic studies on the Aphidiidae. 4. The genera *Pauesia*, *Diaeretus*, *Aphidius* and *Diaeretiella*.. Isr J Entomol.

[29] Kavallieratos NG, Lykouressis DP (1999). Redescription of *Aphidius transcaspicus* Telenga (Hymenoptera: Braconidae) and its distinction from *Aphidius colemani* Viereck (Hymenoptera: Braconidae).. Boll Lab Entomol Agrar Filippo Silvestri.

[30] Kavallieratos NG, Tomanović Ž, Starý P, Athanassiou CG, Sarlis GP (2004). A survey of aphid parasitoids (Hymenoptera: Braconidae: Aphidiinae) of Southeastern Europe and their aphid - plant associations.. Appl Entomol Zool.

[31] Phillips SJ, Anderson RP, Schapire RE (2006). Maximum entropy modeling of species geographic distributions.. Ecol Model.

[32] Hijmans RJ, Cameron SE, Parra JL, Jones PG, Jarvis A (2005). Very high resolution interpolated climate surfaces for global land areas.. Int J Climatol.

[33] Gevrey M, Worner SP (2006). Prediction of global distribution of insect pest species in relation to climate by using an ecological informatics method.. J Econ Entomol.

[34] Zalucki MP, van Klinken RD (2006). Predicting population dynamics of weed biological control agents: science or gazing into crystal balls?. Austral J Entomol.

[35] Gutierrez AP, Ponti L, d'Oultremont T, Ellis CK (2008). Climate change effects on poikilotherm tritrophic interactions.. Climatic Change.

[36] Ulrichs C, Hopper KR (2008). Predicting insect distributions from climate and habitat data.. BioControl.

[37] Kremen C, Cameron A, Moilanen A, Phillips SJ, Thomas CD, Beentje H, Dransfield J, Fisher BL, Glaw F, Good TC, Harper GJ, Hijmans RJ, Lees DC, Louis E, Nussbaum RA, Raxworthy CJ, Razafimpahanana A, Schatz GE, Vences M, Vieites DR, Wright PC, Zjhra ML (2008). Aligning conservation priorities across taxa in Madagascar with high-resolution planning tools.. Science.

[38] Paul JR, Morton C, Taylor CM, Tonsor SJ (2009). Evolutionary time for dispersal limits the extent but not the occupancy of species' potential ranges in the tropical plant genus *Psychotria* (Rubiaceae).. Am Nat.

[39] Phillips SJ, Dudik M (2008). Modeling of species distributions with Maxent: new extensions and a comprehensive evaluation.. Ecography.

[40] Araújo MB, Pearson RG, Thuiller W, Erhard M (2005). Validation of species-climate impact models under climate change.. Global Change Biol.

[41] Wiens JJ (2004). Speciation and ecology revisited: phylogenetic niche conservatism and the origin of species.. Evolution.

[42] Waples RS, Gaggiotti O (2006). What is a population? An empirical evaluation of some genetic methods for identifying the number of gene pools and their degree of connectivity.. Mol Ecol.

[43] Beerli P (2004). Effect of unsampled populations on the estimation of population sizes and migration rates between sampled populations.. Mol Ecol.

[44] Garrigan D, Kingan SB, Pilkington MM, Wilder JA, Cox MP, Soodyall H, Strassmann H, Destro-Bisol G, de Knijff P, Novelletto A, Friedlaender J, Hammer MF (2007). Inferring human population sizes, divergence times and rates of gene flow from mitochondrial, X and Y chromosome resequencing data.. Genetics.

[45] Castella V, Ruedi M, Excoffier L, Ibáñez C, Arlettaz R, Hausser J (2000). Is the Gibraltar Strait a barrier to gene flow for the bat *Myotis myotis* (Chiroptera: Vespertilionidae)?. Mol Ecol.

[46] Gantenbein B, Largiadèr CR (2003). The phylogeographic importance of the Strait of Gibraltar as a gene flow barrier in terrestrial arthropods: a case study with the scorpion *Buthus occitanus* as model organism.. Mol Phylogeneti Evol.

[47] Horn A, Roux-Morabito G, Lieutier F, Kerdelhue C (2006). Phylogeographic structure and past history of the circum-Mediterranean species *Tomicus destruens* Woll. (Coleoptera: Scolytinae).. Mol Ecol.

[48] Schmitt T, Röber S, Seitz A Is the last glaciation the only relevant event for the present genetic population structure of the Meadow Brown butterfly *Maniola jurtina* (Lepidoptera: Nymphalidae)?. Biol J Linn Soc.

[49] Graham CH, Elith J, Hijmans RJ, Guisan A, Peterson AT (2008). The influence of spatial errors in species occurrence data used in distributional models.. J Appl Ecol.

[50] Lozier JD, Roderick GK, Mills NJ (2009). Tracing the invasion history of mealy plum aphid, *Hyalopterus pruni* (Hemiptera: Aphididae), in North America: a population genetics approach.. Biol Invasions.

[51] Warren DL, Glor RE, Turelli M (2008). Environmental niche equivalency versus conservatism: quantitative approaches to niche evolution.. Evolution.

[52] Messenger PS, Wilson F, Whitten MJ, Huffaker CB, Messenger PS (1976). Variation, fitness, and adaptability of natural enemies.. Theory and Practice of Biological Control.

[53] Hopper KR, Roush RT, Powell W (1993). Management of genetics of biological-control introductions.. Annu Rev Entomol.

[54] Hoelmer KA, Kirk AA (2005). Selecting arthropod biological control agents against arthropod pests: Can the science be improved to decrease the risk of releasing ineffective agents?. Biol Control.

[55] Legrand MA, Salin C, Langer A, Hance T (2004). Are mummy characteristics reliable indicators of diapause and cold tolerance in the parasitoid wasp *Aphidius rhopalosiphi* (Braconidae, Aphidiinae)?. Cryo Lett.

[56] Hance T, van Baaren J, Vernon P, Boivin G (2007). Impact of extreme temperatures on parasitoids in a climate change perspective.. Annu Rev Entomol.

[57] Roy HE, Cottrell TE (2008). Forgotten natural enemies: Interactions between coccinellids and insect-parasitic fungi.. Europ J Entomol.

[58] van den Bosch R, Hom R, Matteson P, Frazer BD, Messenger PS, Davis CS (1979). Biological control of the walnut aphid in California – impact of the parasite, *Trioxys pallidus*.. Hilgardia.

[59] Liu SS, Gebremeskel FB, Shi ZH (2002). Reproductive compatibility and variation in survival and sex ratio between two geographic populations of *Diadromus collaris*, a pupal parasitoid of the diamondback moth, *Plutella xylostella*.. BioControl.

[60] Ellegren H (2000). Microsatellite mutations in the germline: implications for evolutionary inference.. Trends Genet.

[61] Brower AVZ (1996). Parallel race formation and the evolution of mimicry in *Heliconius* butterflies: A phylogenetic hypothesis from mitochondrial DNA sequences.. Evolution.

[62] Schug MD, Hutter KA, Wetterstrand KA, Gaudette MS, Mackay TF, Aquadro CF (1998). The mutation rates of di-, tri-, and tetranucleotide repeats in *Drosophila melanogaster*.. Mol Biol Evol.

[63] Hewitt GM (1996). Some genetic consequences of ice ages, and their role in divergence and speciation.. Biol J Linn Soc.

[64] Hewitt GM (2004). Genetic consequences of climatic oscillations in the Quaternary.. Phil Trans Biol Sci.

[65] Schmitt T (2007). Molecular biogeography of Europe: pleistocene cycles and postglacial trends.. Front Zool.

[66] Slatkin M (1993). Isolation by distance in equilibrium and non-equilibrium populations.. Evolution.

[67] Lozier JD, Roderick GK, Mills NJ (2007). Genetic evidence from mitochondrial, nuclear, and endosymbiont markers for the evolution of host plant associated species in the aphid genus *Hyalopterus* (Hemiptera: Aphididae).. Evolution.

[68] Smartt J, Simmonds NW (1995). Evolution of crop plants.

[69] Richards CL, Carstens BC, Knowles LL (2007). Distribution modelling and statistical phylogeography: an integrative framework for generating and testing alternative biogeographical hypotheses.. J Biogeogr.

[70] Waltari E, Hijmans RJ, Peterson AT, Nyári ÁS, Perkins SL (2007). Locating Pleistocene Refugia: Comparing Phylogeographic and Ecological Niche Model Predictions.. PLoS ONE.

