# Supplementary material for: Ecological Niche Models and Coalescent Analysis of Gene Flow Support Recent Allopatric Isolation of Parasitoid Wasp Populations in the Mediterranean
Source: PLoS One. 2009 Jun 12;4(6):e5901. doi: 10.1371/journal.pone.0005901 (PMC2691581; doi:10.1371/journal.pone.0005901)
Supplement: Table S1 — Supplementary coordinate data. Coordinates for presence localities used in the ecological niche models (0.12 MB DOC) [file pone.0005901.s001.doc]

Table S1: Coordinates used in ecological niche model analysis

| Sample name | cluster | Latitude (decimal degrees) | Longitude (decimal degrees) | country |
| --- | --- | --- | --- | --- |
| B14 | K1 | 37.35 | -2.07 | Spain |
| B15 | K1 | 37.36 | -2.04 | Spain |
| B22 | K1 | 38.14 | -0.96 | Spain |
| B23 | K1 | 38.14 | -0.96 | Spain |
| B24 | K1 | 38.23 | -1.08 | Spain |
| B25 | K1 | 38.23 | -1.08 | Spain |
| B26 | K1 | 38.52 | -0.91 | Spain |
| B28 | K1 | 38.6 | -0.68 | Spain |
| B31 | K1 | 38.51 | -0.23 | Spain |
| E01 | K1 | 37.17 | 10.03 | Tunisia |
| E02 | K1 | 37.17 | 10.03 | Tunisia |
| E03 | K1 | 37.01 | 9.88 | Tunisia |
| E04 | K1 | 36.46 | 10.74 | Tunisia |
| E05 | K1 | 36.78 | 10.99 | Tunisia |
| E06 | K1 | 36.7 | 10.49 | Tunisia |
| E07 | K1 | 33.76 | 9.99 | Tunisia |
| E08 | K1 | 36.4 | 10.62 | Tunisia |
| L01 | K1 | 37.52 | -6.344 | Spain |
| L02 | K1 | 37.104 | -6.697 | Spain |
| L03 | K1 | 37.3832 | -6.83375 | Spain |
| L04 | K1 | 37.3832 | -6.83375 | Spain |
| L05 | K1 | 36.222 | -5.459 | Spain |
| L07 | K1 | 36.589 | -5.592 | Spain |
| L08 | K1 | 36.63 | -4.83 | Spain |
| L09 | K1 | 36.872 | -5.014 | Spain |
| L10 | K1 | 36.868 | -4.959 | Spain |
| L11 | K1 | 36.845 | -4.442 | Spain |
| L14 | K1 | 36.85 | -2.95 | Spain |
| M01 | K1 | 35.844 | -5.56 | Morocco |
| M02 | K1 | 35.761 | -5.511 | Morocco |
| M03 | K1 | 35.11 | -5.28 | Morocco |
| M04 | K1 | 35.24 | -3.93 | Morocco |
| M05 | K1 | 34.967 | -2.239 | Morocco |
| M07 | K1 | 35.21 | -5.32 | Morocco |
| F01 | K2 | 40.9 | 17.28 | Italy |
| F06 | K2 | 39.89 | 18.33 | Italy |
| F07 | K2 | 39.91 | 18.19 | Italy |
| F11 | K2 | 40.45 | 17.51 | Italy |
| F13 | K2 | 40.62 | 16.93 | Italy |
| AtEgy1 | K3 | 31.48 | 30.6 | Egypt |
| Cyp5 | K3 | 35.17 | 33.34 | Cyprus |
| D06 | K3 | 35.067 | 25.201 | Greece |
| D08 | K3 | 35.129 | 24.867 | Greece |
| D12 | K3 | 35.274 | 24.536 | Greece |
| D20 | K3 | 37.746 | 21.444 | Greece |
| J01 | K3 | 32.785 | 35.65 | Israel |
| J02 | K3 | 33.074 | 35.589 | Israel |
| J03 | K3 | 33.2 | 35.585 | Israel |
| J04 | K3 | 33.147 | 35.64 | Israel |
| J05 | K3 | 33.084 | 35.239 | Israel |
| J06 | K3 | 33.027 | 35.48 | Israel |
| J07 | K3 | 32.712 | 35.56 | Israel |
| J08 | K3 | 32.56 | 34.97 | Israel |
| J09 | K3 | 31.29 | 35.37 | Israel |
| J10 | K3 | 31.83 | 34.87 | Israel |
| J11 | K3 | 31.81 | 34.91 | Israel |
| K01 | K3 | 35.304 | 25.253 | Greece |
| K02 | K3 | 35.2 | 25.7 | Greece |
| K03 | K3 | 35.067 | 25.201 | Greece |
| K04 | K3 | 35.067 | 25.201 | Greece |
| K05 | K3 | 35.067 | 25.201 | Greece |
| K06 | K3 | 35.067 | 25.201 | Greece |
| K08 | K3 | 35.352 | 24.788 | Greece |
| K09 | K3 | 35.074 | 24.762 | Greece |
| K10 | K3 | 35.02 | 25.76 | Greece |
| K11 | K3 | 35.074 | 24.762 | Greece |
| K12 | K3 | 35.015 | 25.83 | Greece |
| K13 | K3 | 35.015 | 25.83 | Greece |
| K14 | K3 | 35.015 | 25.83 | Greece |
| K15 | K3 | 35.28 | 25.45 | Greece |
| K16 | K3 | 35.28 | 25.45 | Greece |
| AtTurk1 | K3 | 37.8928 | 36.93 | Turkey |
| Kav1 | K3 | 38.3759 | 23.1013 | Greece |
| Kav2 | K3 | 38.3949 | 21.4369 | Greece |
| Kav3 | K3 | 39.23 | 23.22 | Greece |
| AtIrn1 | K4 | 35.814 | 51.41 | Iran |
| AtIrn2 | K4 | 32.75 | 51.63 | Iran |
| AtIrn3 | K4 | 35.814 | 51.41 | Iran |
| AtIrn4 | K4 | 32.75 | 51.63 | Iran |
| AtIrn5 | K4 | 39.95 | 46.88 | Iran |
| AtPak1 | K5 | 33.72 | 73.06 | Pakistan |
